# The burdens of tuberculosis on patients with malignancy: incidence, mortality and relapse

**DOI:** 10.1038/s41598-019-48395-8

**Published:** 2019-08-15

**Authors:** Chin-Chung Shu, Kuang-Ming Liao, Yi-Chen Chen, Jhi-Joung Wang, Chung-Han Ho

**Affiliations:** 1 Department of Internal Medicine, National Taiwan University Hospital, Taipei, Taiwan; 2 College of Medicine, National Taiwan University, Taipei, Taiwan; 3 Department of Internal Medicine, Chi Mei Medical Center, Chiali, Taiwan; 4 Department of Medical Research, Chi Mei Medical Center, Tainan, Taiwan; 5 AI Biomed Center, Southern Taiwan University of Science and Technology, Tainan, Taiwan; 6 Department of Hospital and Health Care Administration, Chia Nan University of Pharmacy and Science, Tainan, Taiwan

**Keywords:** Epidemiology, Tuberculosis

## Abstract

Population with malignancy is growing worldwide; however, its tuberculosis (TB) burden including remains unclear regarding incidence, mortality, and relapse. We retrieved information and identified patients with malignancy and TB between 2000 and 2015 from the Taiwanese National Health Insurance reimbursement datasets, Taiwan cancer registry and death registration. We analyzed the incidence of new TB in patients with malignancy and their mortality as well as TB recurrence. During study period, we reviewed 1,105,009 patients after exclusion and among them, 19,906 had newly diagnosed TB. The TB incidence in cancer patients divided all TB events increased annually, from 3% in 2000 to 13% in 2015. The standard incidence rates (SIR) were highest in cancer of respiratory tract (5.45), hematology (3.70) and then head and neck area (2.58). The mortality directly due to TB was defined as 0.83% and all-cause mortality were approximately 10.5% at 3 months and 20.56% at 12 months. After completing TB treatment, recurrence was diagnosed in 626 (3.14%), and 1001 (5.03%) patients within the first and the first two years, respectively. In conclusion, the incidence of TB in patients with malignancy increase yearly as well as its proportion within overall cases. The twelve-month all-cause mortality during TB and the two-year recurrence are as high as 20.56% and 5.03%, respectively. It indicates the importance of this population in future TB control, especially for those with malignancy of respiratory tract, and hematology as well as head and neck area.

## Introduction

Tuberculosis (TB) remains the most common infectious disease worldwide and leads to high mortality^1^. Currently, one-fourth of the global population has been infected by *Mycobacterium tuberculosis*. According to a World Health Organization (WHO) report, 10.0 million people suffer from active TB infection, and 1.3 million people die from TB every year^2^. For future successful control of TB in the post-2015 era, preventing TB reactivation and transmission in high-risk groups is an important strategy^3^. The WHO suggests the commencement of targeted screening of high-risk groups in high- or upper-middle-income countries with a TB incidence of <100 per 100,000 person-years^4^.

Among the high-risk TB population, patients diagnosed with malignancy need to be targeted because their population is still growing^5–7^. In addition, due to advancements of current medications, the average lifespan after a diagnosis of cancer is longer than before^8^. The risk for TB in patients with malignancy is due to immunosuppression from the cancer itself or from the chemotherapy and local structural changes in the lungs by primary lung cancer or metastasis^9,10^. The incidence of TB has been therefore reportedly increasing in patients with cancer, in both pulmonary and non-pulmonary cancers^11–13^. The risk of TB reactivation reasonably increases in people with cancer, and therefore, screening for active and latent TB in this group should be considered^14^. However, the number of cases of incident TB from previous reports is small^11,13^, and it is unclear what the proportion is for TB infection in cancer patients divided by the overall number of TB patients, which is declining under the current strategy to eliminate TB worldwide^15^. If this proportion increases, the importance of TB in cancer needs to be emphasized more in the strategy to eliminate TB. In addition, the other perspectives of TB-related burdens, including mortality and recurrence, should be investigated for evaluating the prognosis in patients with malignancy; however, these perspectives have rarely been studied. Therefore, we conducted this retrospective study analyzing the nationwide health database to answer these questions in an intermediate TB-prevalent country.

## Methods

### Data source

The Health and Welfare Data Science Center (HWDC) set an integrated database to provide complete information about the Taiwanese National Health Insurance (NHI) reimbursement datasets, Taiwan Cancer Registry (TCR), and death registration for Taiwan population. The NHI reimbursement datasets, which were a single-payer insurance system, consisted of detailed health-care information coverage of more than 99% of Taiwan’s total population and included inpatient and ambulatory care claims from 1996 to 2015. The TCR included information on individual demographics, cancer stages, cancer primary sites, tumor histologies, and treatment types.

For research purposes, the database from the HWDC is released to the public with de-identified forms in an anonymous format. This study was conducted in compliance with the Declaration of Helsinki and has been approved by the Research Ethics Committee of Chi Mei Hospital (IRB no.10803-E01). In addition, the requirement of patient informed consent was waived.

### Definitions of study subjects

**Figure 1:**
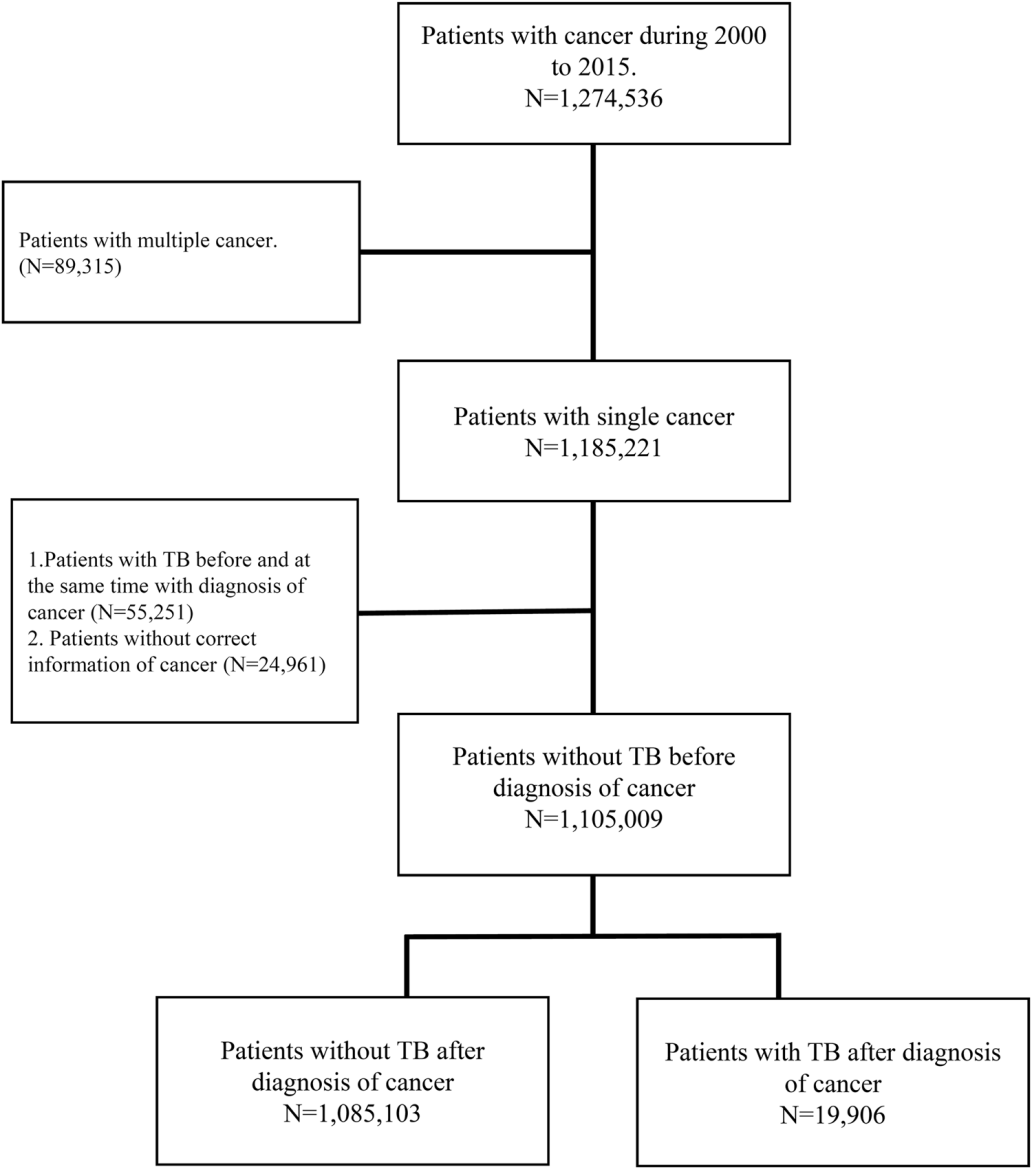
Flow chart of case enrollment. TB, tuberculosis.

The study subjects were selected from the TCR between 2000 and 2015. To reduce the confounding bias, patients diagnosed with multiple cancers and with incomplete information in the TCR were excluded from this study. Since the aim of this study was to identify the new-onset TB incidence risk among cancer patients, TB was identified based on the International Classification of Diseases, Ninth Revision, Clinical Modification (ICD-9-CM) codes 010–012 and 010–018 of at least one admission diagnosis or two times (within 6 months) diagnosis in outpatient clinic using NHI reimbursement datasets. Patients with a history of TB before or within 3 months after the date of cancer registration were excluded from this study. The flowchart of study subjects’ selection is presented in Fig. 1.

### Measurements

The primary outcome of this study was TB. All study subjects were followed up until the onset of new TB infection, death, or the end date of the study, December 31, 2015. According to the International Classification of Diseases for Oncology, third edition (ICD-O-3), the cancer types were divided into head and neck (ICD-O-3: C00-C14), digestive (ICD-O-3: C15-C26 and C48), respiratory (ICD-O-3: C30-C39), bone, skin and soft tissues (ICD-O-3: C40, C41, C44, C47, and C49), breast (ICD-O-3: C50), urinary tract (ICD-O-3: C64-C68), female genital tract (ICD-O-3: C55, C58, C51-C52, and C577-C579), male genital tract and prostate (ICD-O-3: C60-C63), and hematology (ICD-O-3: C81-C96), and other cancers.

The secondary outcomes were mortality after TB and the recurrence of TB. The mortality was based on the death registration database, and the cause of death with TB diagnosed was also recorded using the International Classification of Diseases, Tenth Revision (ICD-10). TB recurrence was identified as patients with a second TB occurrence after completing treatment for a TB infection. According to previous studies, the completion treatment was defined as at least 6 months of treatment; and the last claim date of TB diagnosis with no longer treatment regimens given for more than 3 months^14^.

### Statistical analysis

The frequency with percentage is presented as the incidence number of TB among the cancer population. The incidences of TB (per 100,000 person-years) among patients with different cancer types were calculated. According to the assumption of the Poisson distribution, the standardized incidence ratio (SIR) of TB was estimated using the comparison between the observed incidence in study subjects and expected TB incidence the general population in 2011 (representative year during the study period). Furthermore, to determine the higher risk of TB in specific cancer types, subgroup analyses of different age groups and sex among different cancer types compared with head and neck cancer are also presented using forest plots. All analyses were conducted using SAS statistical software version 9.4 (SAS Institute, Inc., Cary, NC, USA). The statistical significance was set at a p-value < 0.05.

## Results

During 2000–2015, we reviewed 1,274,536 patients with malignancy, and of them, we finally analyzed 1,105,009 patients after excluding those with multiple cancers (n = 89,315), those with a TB infection prior to cancer diagnosis (n = 55,251), and those without correct information of cancer (n = 24,961) (Fig. 1). On average, the enrolled patients with malignancy were aged 65 and had a male proportion of 55%. The malignancy was classified based on the origin as follows: digestive system (35.68%), respiratory tract (11.87%), breast (11.83%), female genital tract (9.73%), head and neck (7.78%), urinary tract (5.01%), bone, skin and soft tissues (4.26%), hematology (4.43%), male genital tract (4.41%), and others (5.00%) (Table 1).

**Table 1:** The crude and standardized incidence rate (SIR) of tuberculosis in different cancer type.

Malignancy, primary location	N (% of total)	Person-year	Event	Rate*	SIR^#^ (95% C.I.)
**Total**	1,105,009 (100.00)	6383055.73	19,906	312	1.31 (1.30–1.33)
Head and neck	85,950 (7.78)	521311.01	1,990	382	2.58 (2.37–2.80)
Digestive	394,258 (35.68)	1981200.05	5,990	302	1.50 (1.39–1.61)
Respiratory	131,116 (11.87)	543125.91	4,842	892	5.45 (4.92–5.97)
Bone, skin and soft tissues	47,119 (4.26)	306022.29	1,012	331	1.68 (1.50–1.86)
Breast	130,717 (11.83)	907147.77	840	93	0.85 (0.75–0.95)
Urinary tract	55,316 (5.01)	343241.16	1,155	336	1.85 (1.50–2.20)
Female genital tract	107,556 (9.73)	877298.75	824	94	0.83 (0.76–0.90)
Male genital tract and prostate	48,773 (4.41)	305098.64	1,359	445	1.29 (1.06–1.51)
Hematology	49,002 (4.43)	271294.65	1,327	489	3.70 (3.46–3.93)
Other cancer	55,202 (5.00)	327314.50	567	173	1.59 (1.45–1.74)

^*^Rate: per 100,000 person years.

^#^Standardized year 2011.

### TB incidence in patients with malignancy

**Figure 2:**
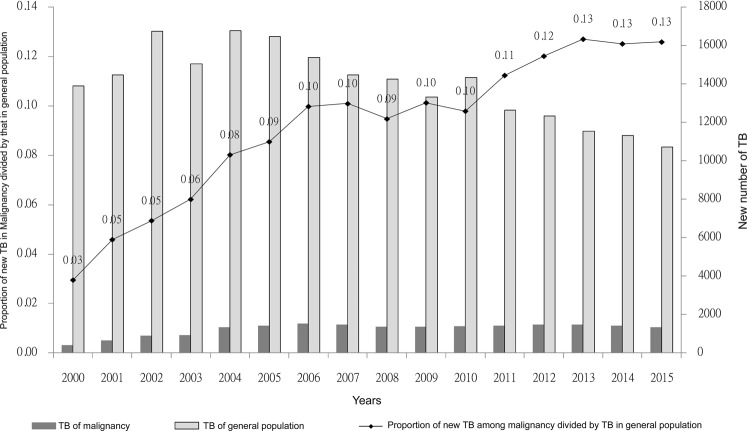
Proportion of new diagnosed tuberculosis between patients with malignancy and general population.

According to the cohort in the database, 19,906 newly diagnosed TB infections after cancer diagnosis were recorded in patients with malignancy. The overall TB incident number decreased yearly since 2006 under the national strategy of “Stop TB” and treatment with “direct observation therapy”, but the TB number from patients with malignancy remained constant (Fig. 2). The proportion of TB incidences from patients with malignancy divided by the overall TB incident number annually increased, from 3% in 2000 to 9% in 2005, 10% in 2010, and 13% in 2015. This result shows the importance of emphasizing the TB burden in malignancy.

The crude incidences of TB, calculated by TB event divided by the follow-up period, were highest in those with respiratory tract cancer (892 per 100,000 person-years) and then in those with hematological malignancy (489 per 100,000 person-years). The SIRs, which were corrected using the national data from 2011, found that all kinds of malignancy led to a higher ratio of TB except for breast cancer (SIR: 0.85, 95% C.I. [0.75–0.95]) and cancer of the female genital tract (SIR: 0.83, 95% C.I. [0.76–0.90]) (Table 1). The highest SIR for TB was due to respiratory tract cancer (5.45 [4.92–5.97]), hematology malignancy (3.70 [3.46–3.93]) and head and neck cancer (2.58 [2.37–2.80]). The other kinds of malignancies had significant SIRs of 1~2-fold. Figure S1 was the risk of tuberculosis in each cancer compared with head and neck cancer according to the sex and age strata. Patients with cancer of respiratory tract and hematological malignancy seemed have higher risk of TB than those with head-and-neck cancer regardless age and sex. By contrast, patients with other cancers led lower risk of TB except middle-aged female with urinary tract cancer had comparable risk.

### Mortality during TB event in patients with malignancy

Among patients with TB after diagnosis of cancer, mortality directly due to TB was defined in 166 patients (0.83%) by matching national cause-of-death data. Table 2 showed mortality of different cancer patients with TB. Patients with head and neck, bone and soft tissue, and urinary tract cancers had more death caused directly by TB. For all-cause mortality, the overall mortality rates were approximately 10.5% at 3 months, 15.56% at 6 months and 20.56% at 12 months after TB. By contrast, general cancer patients without TB had higher all-cause mortality of 5.28% at 3 months, 8.22% at 6 months, and 11.84% at 12 months (all p < 0.001) in comparing with cancer patient with TB (Table S1 in the Supplement File).

**Table 2:** Mortality of different cancer patients with tuberculosis (TB).

Malignancy, primary location	TB after malignancy, N	TB as cause of death^a^, N (%)	Death after TB diagnosis, N (%)		
			Within 3 months	Within 6 months	Within 12 months
**Total**	**19,906**	**166 (0.83%)**	**2090 (10.50%)**	**3096 (15.56%)**	**4092 (20.56%)**
Head and neck	1,990	26 (1.31%)	224 (11.26%)	307 (15.43%)	396 (19.90%)
Digestive	5,990	46 (0.77%)	621 (10.37%)	899 (15.01%)	1165 (19.45%)
Respiratory	4,842	20 (0.41%)	654 (13.51%)	1065 (22.00%)	1460 (30.15%)
Bone, skin and soft tissues	1,012	22 (2.17%)	83 (8.21%)	118 (11.66%)	155 (15.32%)
Breast	840	3 (0.36%)	33 (3.93%)	41 (4.88%)	58 (6.90%)
Urinary tract	1,155	15 (1.30%)	93 (8.05%)	132 (11.43%)	170 (14.72%)
Female genital tract	824	5 (0.61%)	40 (4.85%)	59 (7.16%)	79 (9.59%)
Male genital tract and prostate	1,359	13 (0.96%)	96 (7.06%)	128 (9.42%)	163 (12.00%)
Hematology	1,327	8 (0.60%)	159 (11.98%)	222 (16.73%)	302 (22.76%)
Other cancer	567	8 (1.41%)	87 (15.34%)	125 (22.05%)	144 (25.40%)

^a^With TB diagnosed in the Cause Of Death Data.

Of the different kinds of malignancy, a higher death rate was observed in patients with respiratory tract cancer (13.51% at 3 months; 30.15% at 12 months) and hematology malignancy (11.98% at 6 months; 22.76% at 12 months). In contrast, breast cancer and cancer from the female genital tract correlated with a lower 12-month all-cause death rate. Comparing with respiratory cancer, the two-year mortality risk is 0.23 fold lower in patients with female genital tract and breast cancer when they got TB. (Table S2 in the Supplement File).

### TB recurrence in patients with malignancy

After completing treatment for TB, recurrence in patients with malignancy was recorded 3 months later. There were 626 (3.14%), 375 (1.88%), and 1001 (5.03%) patients with TB recurrence during 3~12, 12~24, and 3~24 months, respectively, after completing treatment for TB. Table 3 showed TB recurrence according to different malignancy. The recurrence rate of TB during 3~24 months after completing treatment for TB was high in patients with female genital tract cancer (7.28%), breast cancer (7.26%) and male genital tract and prostate cancers (6.18%). In contrast, patients with cancer in the respiratory tract (4.23%) or the digestive system (4.64%) had a relatively low recurrence rate. Among them, we found the survival time within two years after treatment completion of TB was longer in the female genital and breast cancer than that in respiratory tract cancer. (Table S3 in the Supplement File).

**Table 3:** Tuberculosis (TB) recurrence according to different malignancy.

Malignancy	TB after malignancy, N	TB recurrence after treatment completion N (%)		
		3~12 months	12~24 months	3~24 months
**Total**	**19,906**	**626 (3.14%)**	**375 (1.88%)**	**1001 (5.03%)**
Head and neck	1,990	60 (3.02%)	45 (2.26%)	105 (5.28%)
Digestive	5,990	166 (2.77%)	112 (1.87%)	278 (4.64%)
Respiratory	4,842	145 (2.99%)	60 (1.24%)	205 (4.23%)
Bone, skin and soft tissues	1,012	30 (2.96%)	22 (2.17%)	52 (5.14%)
Breast	840	33 (3.93%)	28 (3.33%)	61 (7.26%)
Urinary tract	1,155	40 (3.46%)	23 (1.99%)	63 (5.45%)
Female genital tract	824	31 (3.76%)	29 (3.52%)	60 (7.28%)
Male genital tract and prostate	1,359	56 (4.12%)	28 (2.06%)	84 (6.18%)
Hematology	1,327	53 (3.99%)	18 (1.36%)	71 (5.35%)
Other cancer	567	12 (2.12%)	10 (1.76%)	22 (3.88%)

## Discussion

In the present study, TB in patients with cancer accounted for an increasing proportion of the annual overall incident TB cases, indicating its importance in the strategy of eliminating TB in the intermediate prevalent areas. The crude incidence and SIR of TB were both higher in patients with respiratory cancer or hematological malignancy than patients with other malignancies. The mortality directly due to TB was only 0.83%, but during TB events, the all-cause mortality increased to 20.56% within 12 months. Even after completing the treatment for TB, the recurrence rate in this population with malignancy was as high as 5.03% within 2 years.

In the last decade, TB prevention in Taiwan has followed “The Global Plan To Stop TB 2006–2015”. The TB incidence in Taiwan has decreased by approximately 40–50% and is in an intermediate TB status. In regard to the end TB strategy in the post-2015 era^15^, screening for active and latent TB infections in high-risk groups is a pivotal strategy. In particular, TB infection in people with cancer has increased from 3% to 13% of all TB cases in intermediate TB areas. This increased proportion means that a further strategy that targets the population with malignancy is needed. In particular, the cancer population is enlarging due to the increasing incidence of cancer and prolonged lifespan after a diagnosis of cancer. A focus on survey for latent TB infection in cancer groups is suggested^4^, but paucity literature with large TB in this population has been reported^11,13^. The present study, to the best of our knowledge, is one of the largest population studies, and includes 19,906 patients with a TB event after the diagnosis of cancer. The results will be helpful in understanding the TB burden in this population.

For the risk of TB in the cancer population, the highest risk was crude incidence of 892 and 489 per 100,000 person-years in patients with respiratory tract cancer and hematological malignancy, respectively, which are traditionally known TB-susceptible populations^14^. After standardizing by age and sex, the SIRs of 5.45, 3.70, and 2.58 were caused by cancer of the respiratory tract, hematology, and head and neck, respectively. The risks of TB in these cancers are compatible with previous meta-analyses and show that these patient populations should be a priority for TB screening^13^. In regard to breast and female genital cancers, SIRs showed that these cancers were unexpectedly protective for TB. There are several possible explanations for this result. First, the incidence of TB from these cancers is not relatively high, and some studies even show no significant increase in the risk for TB infection^13,16,17^. Second, this study did not discriminate the risk from different cancer statuses. Under the current screening strategies for breast and cervical cancers, the cancer could be detected at an early stage and could be cured or kept stable. Therefore, the current screening strategy might be correlated with the low risk for TB infection. In addition, these patients are instructed to protect themselves from infection and could have decreased risks of TB transmission. Third, the female sex is associated with a lower tendency of developing TB than that of the male sex^18–21^, and the crude incidence in the present study might be lower in female-specific cancers than in male-specific cancers.

In addition to the occurrence rate, mortality, either directly or indirectly by TB during the infection, is another important issue considered in the prevention strategy. Because years of lost life is a major concern in evaluating the cost-effectiveness of the strategy. We used the national cause-of-death database and found that the incidence of TB directly related to mortality was approximately 0.83% in the cancer population, which is higher than 0.28% in the general population^22^. On the other hand, the all-cause mortality rate during 6 months (15.56%) after the diagnosis of TB was higher than that within 6–12 months (5.0%). In addition, the cancer patients with TB had higher all-cause mortality of 20.56% at 12 months than 11.84% in those without TB, indicating that active TB infection correlated with a worse outcome in patient with malignancy. Previous studies have shown that preexisting TB is associated with an increased mortality in lung cancer^23,24^. However, the influence of active TB on cancer has rarely been previously reported^25^. The mortality in different cancers in this analysis showed that respiratory tract, hematology and head and neck cancers had the highest all-cause mortality, suggesting their weak host and tendency to be threatened by infection. In contrast, breast and female genital cancers had the lowest all-cause mortality. This result might be due to the prognosis of different cancer type. For example, lung cancer leads higher mortality by itself^26,27^. In addition, TB happens mostly at lung, and possibly exacerbates respiratory compromise in patients with respiratory tract cancer because their lung reserve is lower than that in other cancers.

After completing treatment for TB infection, we monitored TB recurrence in patients with malignancy. The recurrence rate is, on average, approximately 3.14% during 3~12 months and 1.88% during 12~24 months after TB treatment completion. The recurrence rate has been rarely reported before and in the present study is higher than the 2-year recurrence rate of 1.38% in the general population who does not have diabetes^28^. Breast and female genital cancers were associated with a higher 2-year recurrence rate and the possible explanation is low mortality in female genital and breast cancer and makes the longer time for potential recurrence (immortal-related bias) (Table S2 and S3 in the Supplement File). For example, our results indicated that patients with respiratory cancer had about 4–5-fold mortality compared than female genital tract and breast cancer patients (Table S2 in the Supplement File). In addition, the mean survival time in female genital tract and breast cancer patients with TB was longer than that in respiratory cancer patients with TB (Table S3 in the Supplement File). Thus, this might be the major reason that leads a higher recurrence rate in female genital and breast cancer.

There are several limitations in the present study. First, it is a retrospective study design, and some details such as cause of death and underlying status could not be obtained. Second, we did not use age- and sex-matched controls but instead compared the results with national average TB data. Third, the detailed risks of TB in different cancer statuses were not investigated in this study. The validation in other database or other country’s nationwide database needs to be performed in the future research.

In conclusion, the proportion of TB cases in patients with cancer increased in contrast to the overall decrease in case number in an intermediate prevalent area where the latent tuberculosis infection (LTBI) strategy is needed for high-risk groups. SIRs were high in respiratory tract cancer, hematological malignancy and head and neck cancer. The 12-month all-cause mortality during TB infection is as high as 20.56%, and the 2-year recurrence rate is 5.03% after treating for TB infection. The TB burden remains high in patients with malignancy and should be suggestively prevented in future public strategies.

### Ethics approval and consent to participate

The Research Ethics Committee of Chi Mei Medical Center approved this study (IRB No.: no.10803-E01). The requirement of patient informed consent was waived.

## Supplementary information

Supplement file
