# Supplementary material for: The burdens of tuberculosis on patients with malignancy: incidence, mortality and relapse
Source: Sci Rep. 2019 Aug 15;9:11901. doi: 10.1038/s41598-019-48395-8 (PMC6695428; doi:10.1038/s41598-019-48395-8)
Supplement: Supplementary file 1 — Supplement file [file 41598_2019_48395_MOESM1_ESM.docx]

**Supplemental File**

Title

**The burdens of tuberculosis on patients with malignancy: incidence, mortality and relapse**

Chin-Chung Shu^1,2*^, Kuang-Ming Liao^3*^, Yi-Chen Chen^4^, Jhi-Joung Wang^4,5^, Chung-Han Ho^4,6^

**Institutions:**

^1^Department of Internal Medicine, National Taiwan University Hospital, Taipei, Taiwan

^2^College of Medicine, National Taiwan University, Taipei, Taiwan

^3^Department of Internal Medicine, Chi Mei Medical Center, Chiali, Taiwan

^4^Department of Medical Research, Chi Mei Medical Center, Tainan, Taiwan

^5^AI Biomed Center, Southern Taiwan University of Science and Technology, Tainan, Taiwan

^6^Department of Hospital and Health Care Administration, Chia Nan University of Pharmacy and Science, Tainan, Taiwan

**Corresponding authors**:

Chung-Han Ho, Ph.D.

Department of Medical Research, Chi Mei Medical Center, Tainan, Taiwan

Address: No 901, Zhonghua Road, Yongkang District, Tainan 710, Taiwan

Email: ho.c.hank@gmail.com

*These two authors contributed equally to this work.

**Running title:** TB in patients with malignancy

**Figure S1**. The risk of tuberculosis in each cancer compared with head and neck cancer according to the sex and age strata. (A) cancer of digestive system, respiratory tract, and bone, skin & soft tissue; (B) cancer from urinary tract, hematological, and others; (C) cancer of breast, female and male genital tract.

(A)


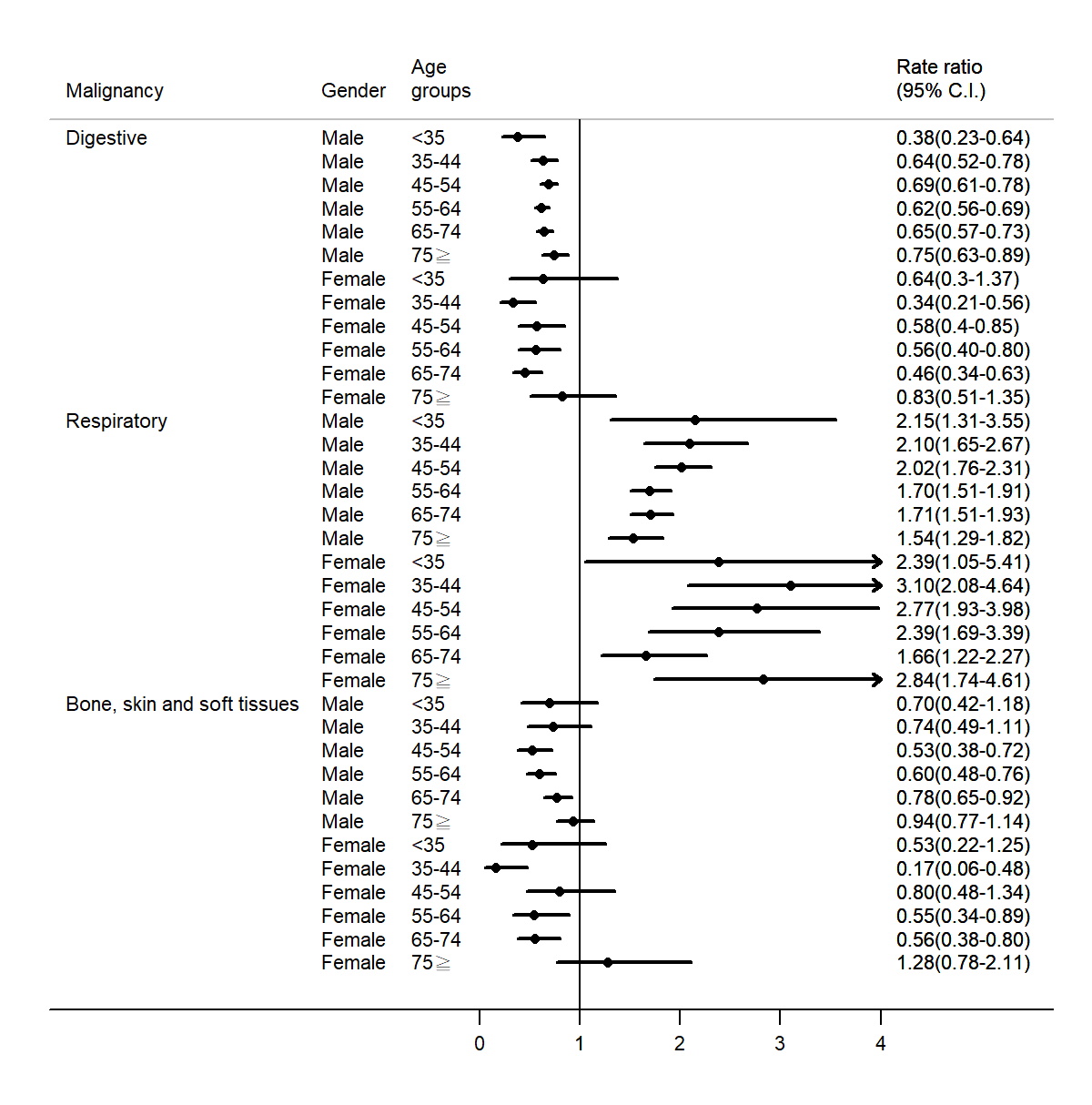


(B)


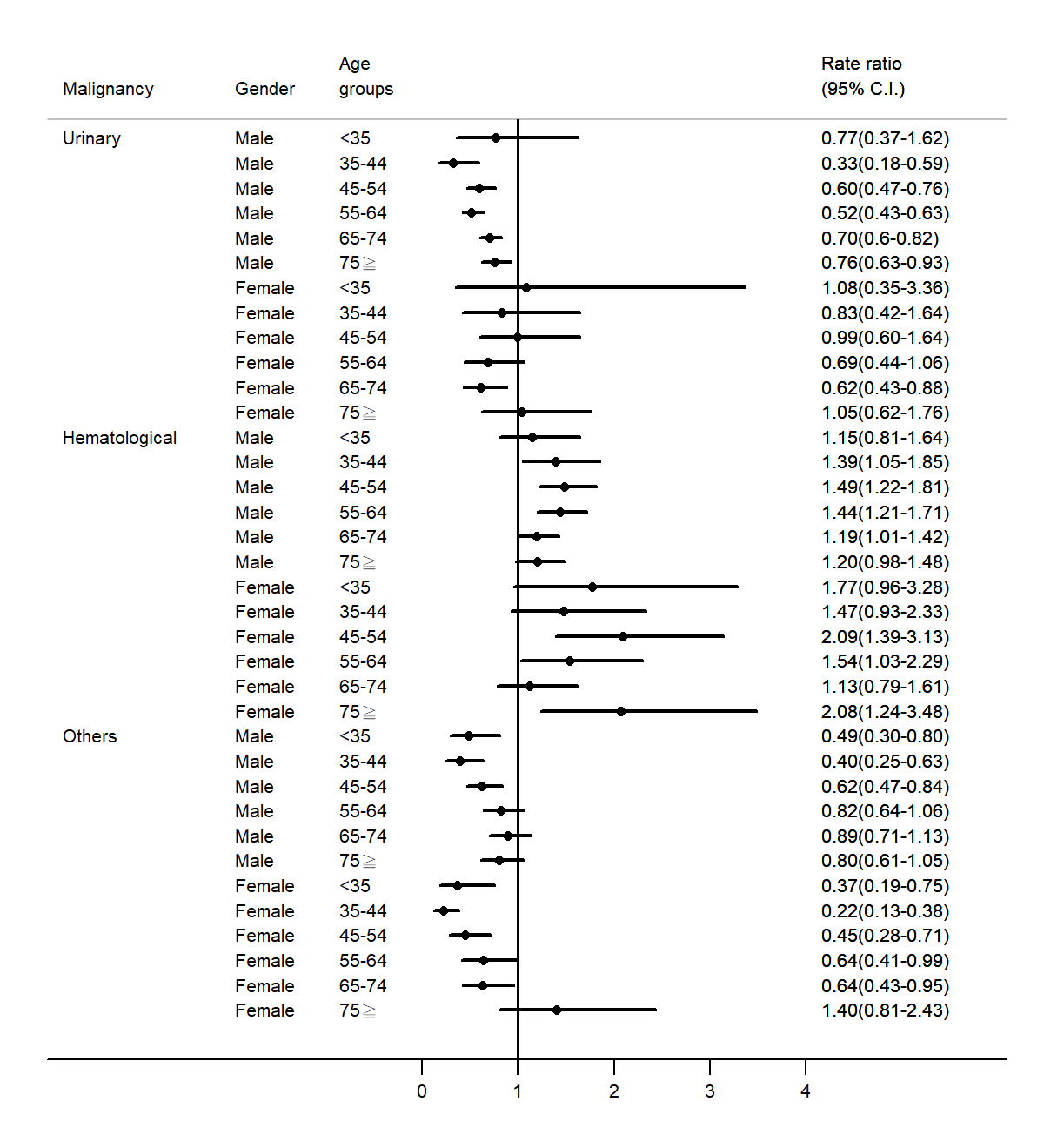


(C)


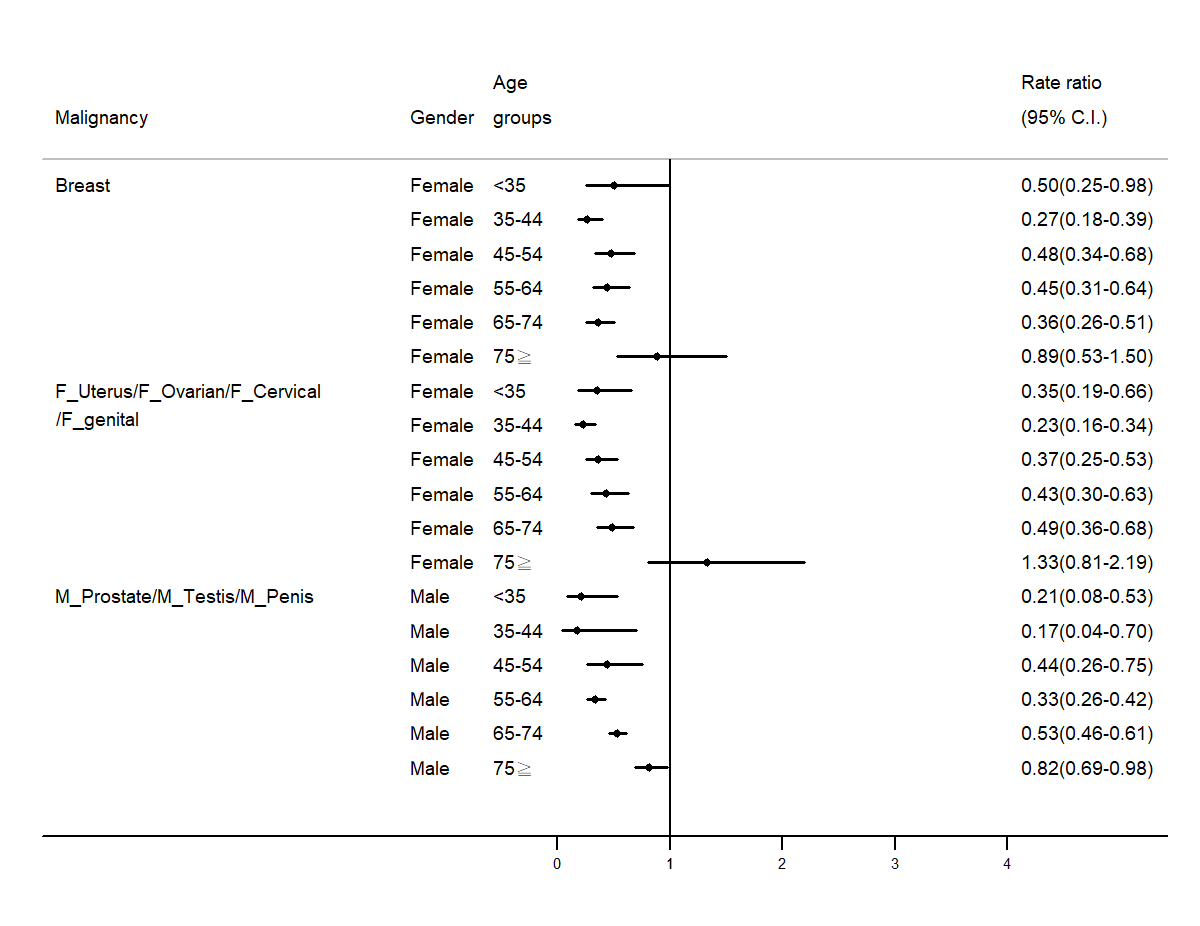


**Table S1.** All-cause mortality rate in cancer patients with or without development of tuberculosis (TB).

| **All-cause mortality rate** | Without TB | With TB | P-value |
| --- | --- | --- | --- |
|  | N=1,085,103 | N=19,906 |  |
| **Overall till the end of study** | 548,255 (50.53%) | 14,461 (72.65%) | <0.0001 |
| **Within 3 months after TB** | 57278 (5.28%) | 2090 (10.50%) | <0.0001 |
| **Within 6 months after TB** | 89238 (8.22%) | 3096 (15.56%) | <0.0001 |
| **Within 12 months after TB** | 128445 (11.84%) | 4092 (20.56%) | <0.0001 |

*The overall all-cause mortality was defined by following up until the end of study date.

**Table S2**. Two-year mortality of tuberculosis (TB) patients under different primary malignancy.

| **Malignancy, primary location** | **N** | **Person-year** | **Death** | **Rate ratio**  **(95% C.I.)** |
| --- | --- | --- | --- | --- |
| Respiratory tract cancer | 4,842 | 4909.97 | 3,341 | Ref. |
| Female genital tract and breast cancer | 1,664 | 2685.76 | 426 | 0.23*(0.18-0.30) |

*P-value<0.001

**Table S3**. Baseline information of tuberculosis (TB) recurrence according to different malignancy with primary TB.

|  | Female genital tract and breast cancer N=1,664 | Respiratory tract cancer, female  N=1,256 | Respiratory tract cancer, male  N=3,586 | P-value |
| --- | --- | --- | --- | --- |
| Two-year mortality, n (%) | 426(25.60) | 729(58.04) | 2612(72.84) | <0.0001 |
| Time to survival, years |  |  |  |  |
| Mean±SD | 1.61±0.67 | 1.17±0.78 | 0.95±0.76 | <0.0001 |
| Recurrence TB, n(%) | 121(7.27) | 63(5.02) | 142(3.96) | <0.0001 |
| Time to recurrence, years |  |  |  |  |
| Mean±SD | 1.04±0.42 | 0.84±0.35 | 0.88±0.36 | 0.0004 |
| CCI group* |  |  |  | 0.0048 |
| 0 | 35(28.93) | 20(31.75) | 26(18.31) |  |
| 1-2 | 48(39.67) | 18(28.57) | 50(35.21) |  |
| 3-6 | 30(24.79) | 10(15.87) | 38(26.76) |  |
| >6 | 8(6.61) | 15(23.81) | 28(19.72) |  |
| Comorbidities |  |  |  |  |
| Chronic pulmonary disease | 40(33.06) | 19(30.16) | 72(50.70) | 0.0028 |
| Diabetes | 30(24.79) | 11(17.46) | 33(23.24) | 0.5191 |
| Liver disease | 16(13.22) | 10(15.87) | 15(10.56) | 0.5510 |
| Inpatients, n(%) | 42(34.71) | 33(52.38) | 92(64.78) | <0.0001 |
| Frequency of IPD |  |  |  |  |
| Median(Q1-Q3) | 2(1-4) | 2(1-5) | 3(2-5) | 0.2190 |
| Frequency of OPD |  |  |  |  |
| Median(Q1-Q3) | 36(23-52) | 41(18-59) | 37(23-59) | 0.8566 |

*Charlson comorbidity index (CCI) was recorded under the inpatient diagnosis for one time or outpatient diagnosis for two times during the one year before recurrence TB and the weight of score was used based on Quan et al. (2011).^1^ The CCI group was categorized the overall sum under each weight to achieve an aggregate score except malignancy.

Reference

1 Quan, H. *et al.* Updating and validating the Charlson comorbidity index and score for risk adjustment in hospital discharge abstracts using data from 6 countries. *Am J Epidemiol* **173**, 676-682
